# Supplementary material for: Reconstructed Ir‒O‒Mo species with strong Brønsted acidity for acidic water oxidation
Source: Nat Commun. 2023 Jul 12;14:4127. doi: 10.1038/s41467-023-39822-6 (PMC10338439; doi:10.1038/s41467-023-39822-6)
Supplement: Supplementary file 1 — Supplementary Information [file 41467_2023_39822_MOESM1_ESM.pdf]

Supplementary Information

Reconstructed Ir–O–Mo species with strong Brønsted acidity for acidic water oxidation

Shiyi Chen[a,1], Shishi Zhang[a,1], Lei Guo[a], Lun Pan[a,b], Chengxiang Shi[a,b], Xiangwen Zhang[a,b], Zhen-Feng Huang[a,b,*], Guidong Yang[c,*] and Ji-Jun Zou[a,b,*]

[a]Key Laboratory for Green Chemical Technology of the Ministry of Education, School of Chemical Engineering and Technology, Tianjin University, Tianjin 300072, China; Collaborative Innovative Centre of Chemical Science and Engineering (Tianjin), Tianjin 300072, China.

[b]Haihe Laboratory of Sustainable Chemical Transformations, Tianjin 300192, China.

[c]XJTU-Oxford International Joint Laboratory for Catalysis, School of Chemical Engineering and Technology, Xi'an Jiaotong University, Xi'an, Shaanxi, China.

*Corresponding author. E-mail address: jj_zou@tju.edu.cn (J.-J. Zou) & zfhuang@tju.edu.cn (Z.-F. Huang) & guidongyang@mail.xjtu.edu.cn (G. Yang)

[1]Shiyi Chen and Shishi Zhang contribute this work equally.

1. Supplementary Figures

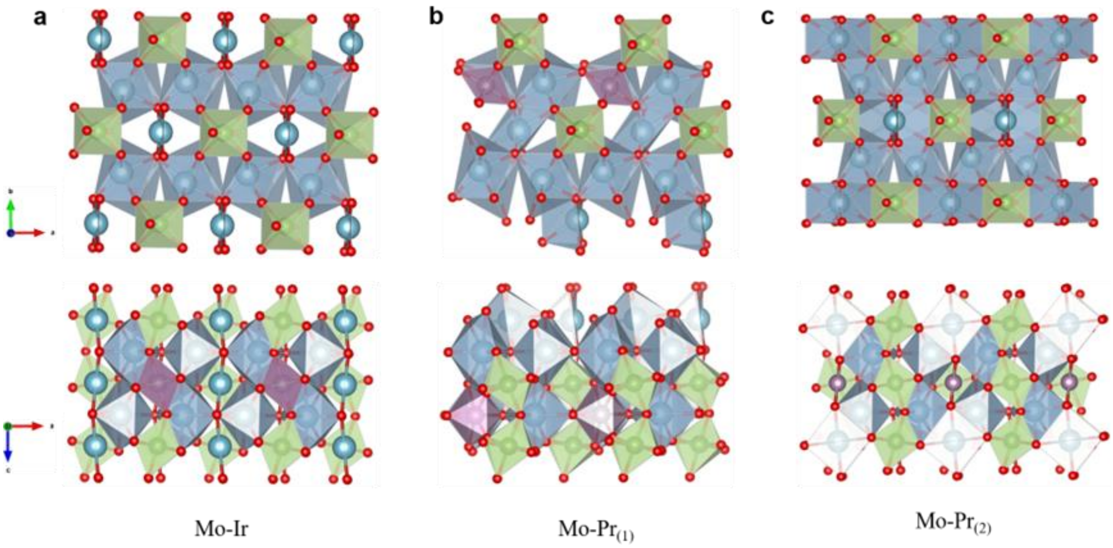

Figure S1. Schematic illustration for the incorporation of Mo at Ir site (a), $Pr_{(1)}$ site (b) and $Pr_{(2)}$ site (c).

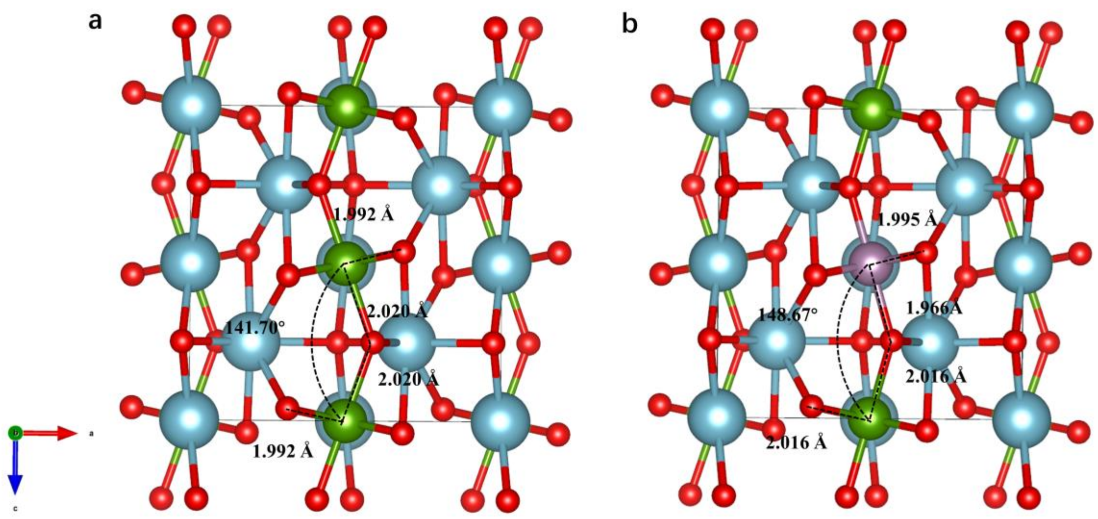

Figure S2. (a), (b) Corresponding bond length and bond angle of Pr_3IrO_7 (a) and Mo-doped Pr_3IrO_7 (b)

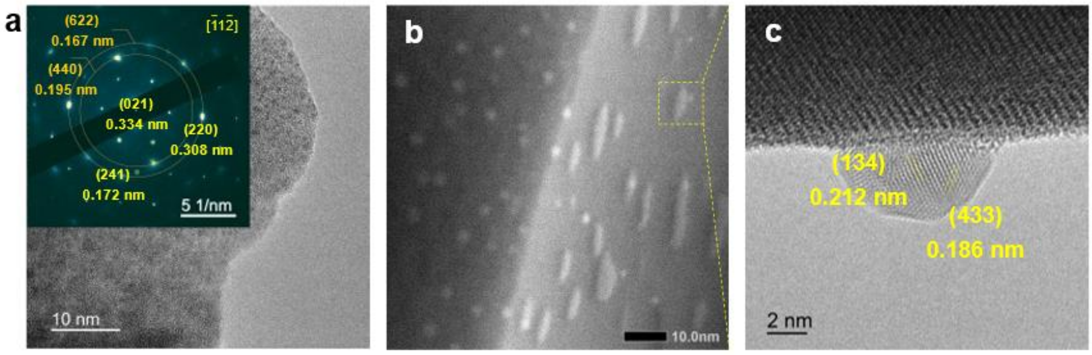

Figure S3. Praseodymium oxides impurities introduced by excess substitution. (a) TEM image of 0.4Mo-PIO. Insets: corresponding SAED patterns. Except for the diffraction pattern from Pr_3IrO_7, another set of diffraction patterns is observed which can be attributed to $Pr_2O_{3.33}$ from XRD results at the near surface region. (b) Secondary-electron (SE) image in the scanning transmission electron microscopy (STEM) mode at the near surface area. (c) HRTEM image of the particles observed from the SE image. The labeled interplanar spacing correspond to (134) and (433) facets of $Pr_2O_{3.33}$.

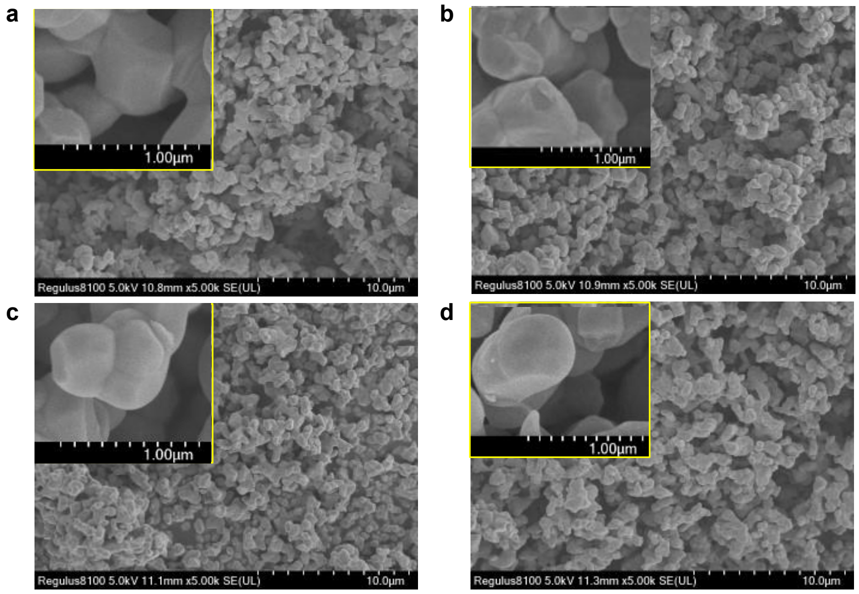

Figure S4. SEM images of the as-synthesized *x*Mo-PIO. (a) x = 0.0 (b) x = 0.1 (c) x = 0.2 (d) x = 0.4.

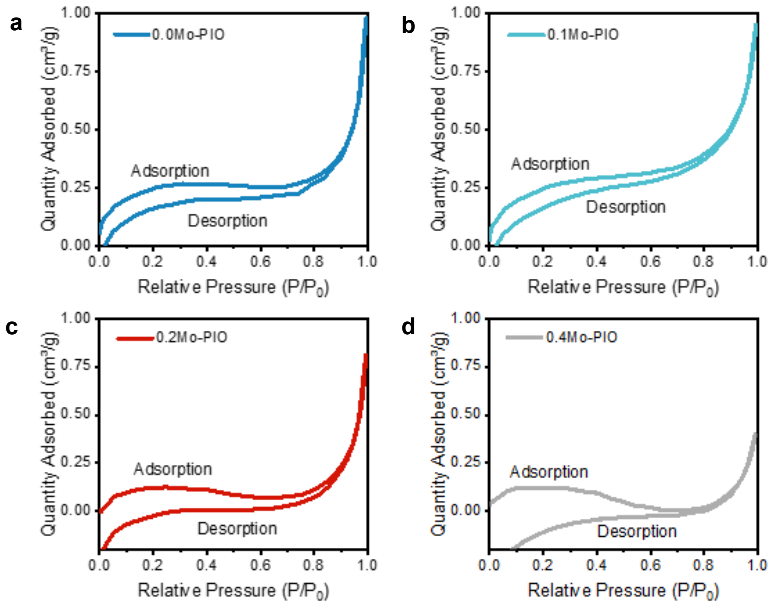

Figure S5. Nitrogen adsorption/desorption isotherms of as-synthesized *x*Mo-PIO samples. (a) $x = 0.0$ (b) $x = 0.1$ (c) $x = 0.2$ (d) $x = 0.4$.

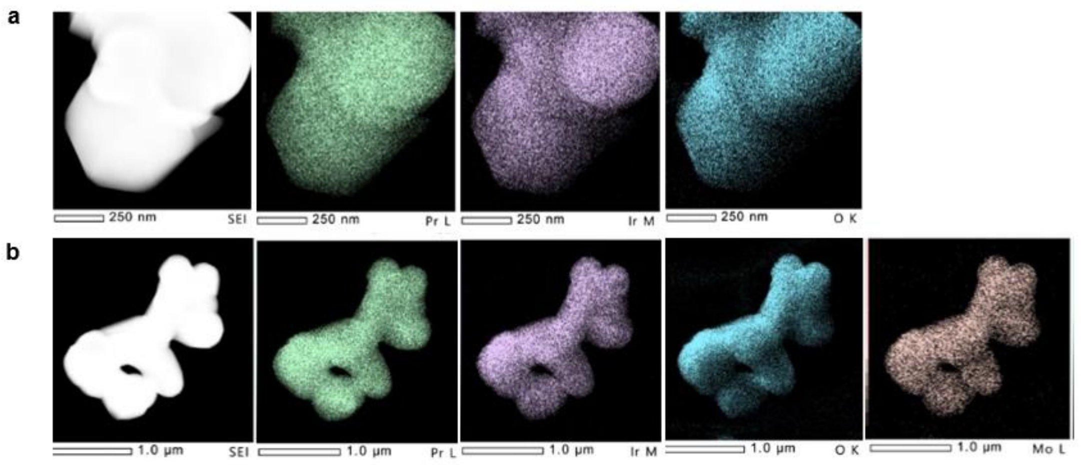

Figure S6. EDS elemental mappings of Pr, Ir, Mo, and O elements for 0.0Mo-PIO (a) and 0.2Mo-PIO (b).

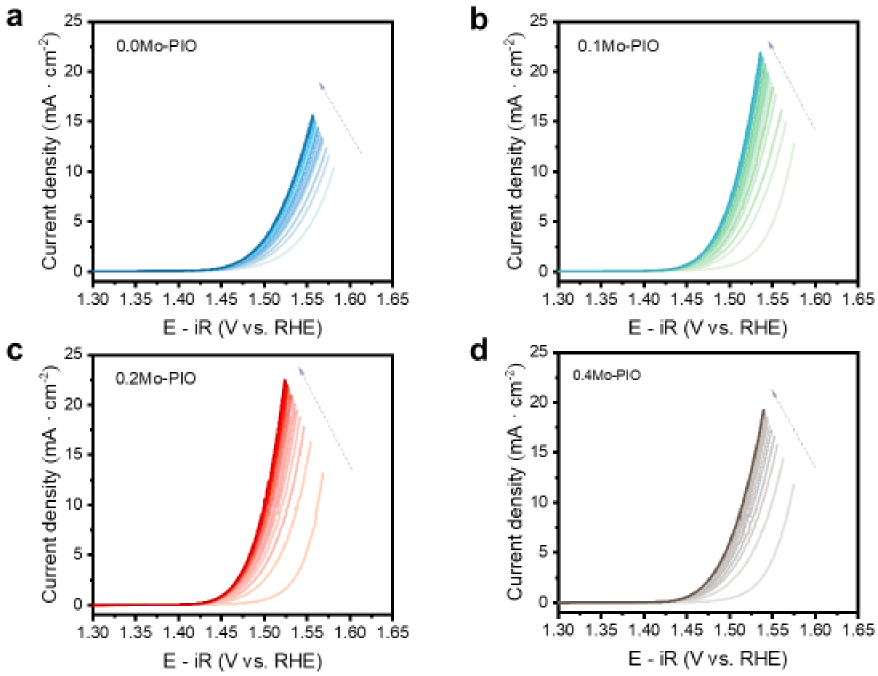

Figure S7. The evolution of LSV curves for *x*Mo-PIO from 1st to the 20th scans in 0.1M $HClO_4$ at 5mV s^{-1}. (a) $x = 0.0$ (b) $x = 0.1$ (c) $x = 0.2$ (d) $x = 0.4$.

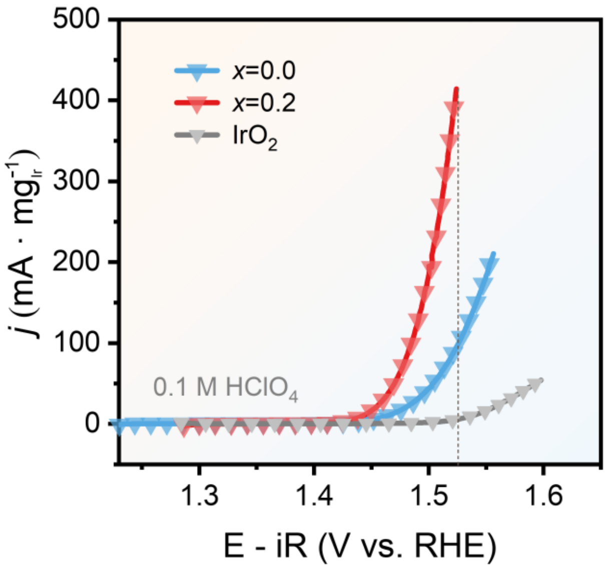

Figure S8. Mass loading-normalized LSV curves of *x*Mo-PIO-post (*x* = 0.0, 0.2) and commercial IrO_2 in 0.1 M $HClO_4$.

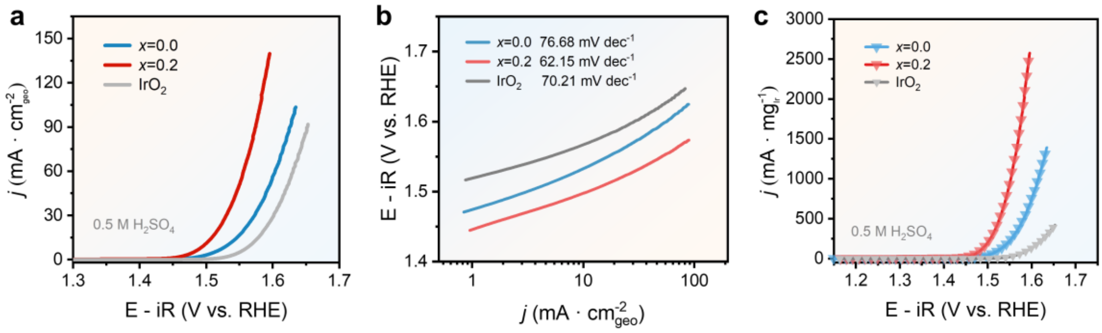

Figure S9. OER performance tests of *x*Mo-PIO-post (*x* = 0.0, 0.2) and commercial IrO_2 conducted in 0.5 M H_2SO_4. (a) Geometric area-normalized LSV curves. (b) Tafel plots based on geometric area-normalized LSV curves. (c) Mass loading-normalized LSV curves.

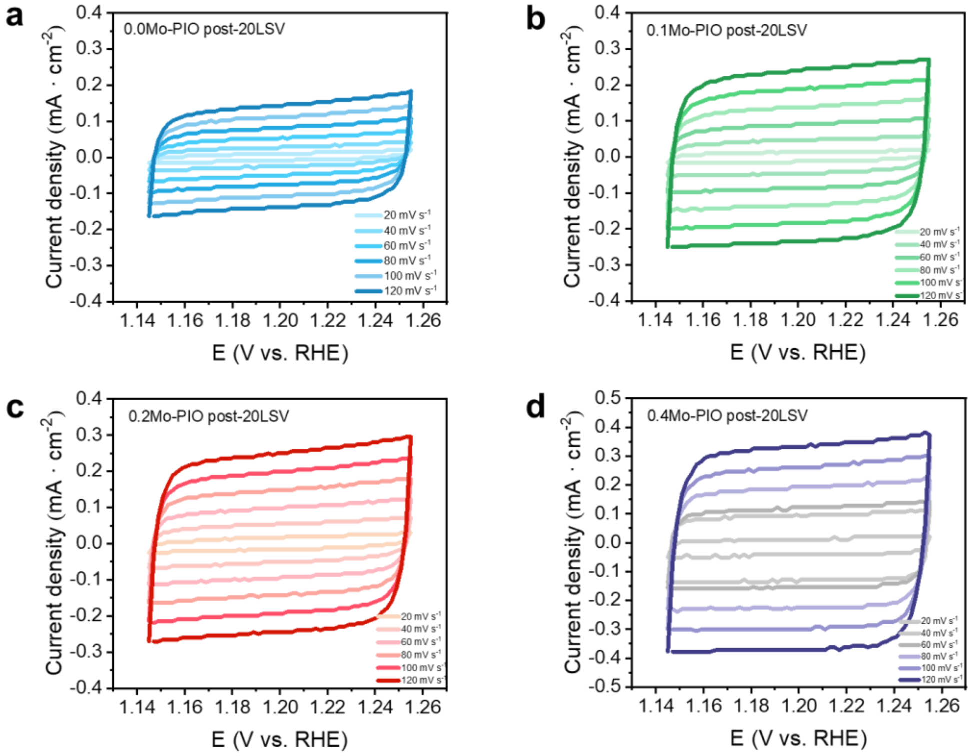

Figure S10. Cyclic voltammograms in the non-Faradaic potential region for *x*Mo-PIO-post in 0.1M $HClO_4$ at different scan rates (20, 40, 60, 80, 100, 120 mV s^{-1}), x = 0.0 (a), 0.1 (b), 0.2 (c), 0.4 (d).

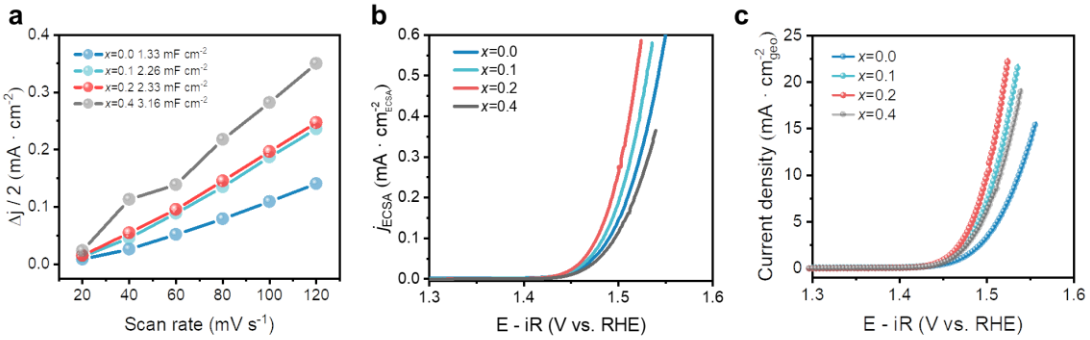

Figure S11. (a) The estimated C_{dl} values from cyclic voltammogram results of *x*Mo-PIO-post (*x* = 0.0, 0.1, 0.2, 0.4). (b) ECSA-normalized and (c) Geometric area-normalized LSV curves of *x*Mo-PIO-post (*x* = 0.0, 0.1, 0.2, 0.4).

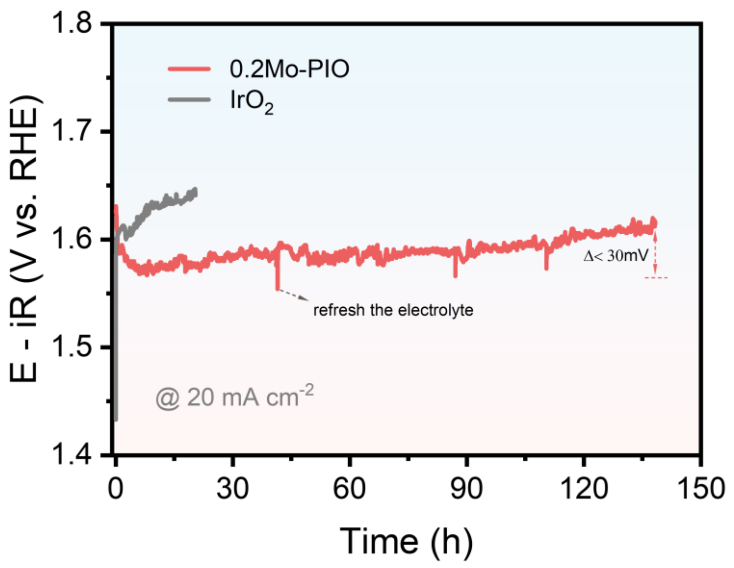

Figure S12. Chronopotentiometry curves of 0.2Mo-PIO and commercial IrO_2 at 20 mA cm^{-2} in 0.1 M $HClO_4$ electrolyte. The abrupt changes of voltage are caused by refreshing the electrolyte and the jagged voltage fluctuation is due to the release of bubbles accumulating on the electrode surface.

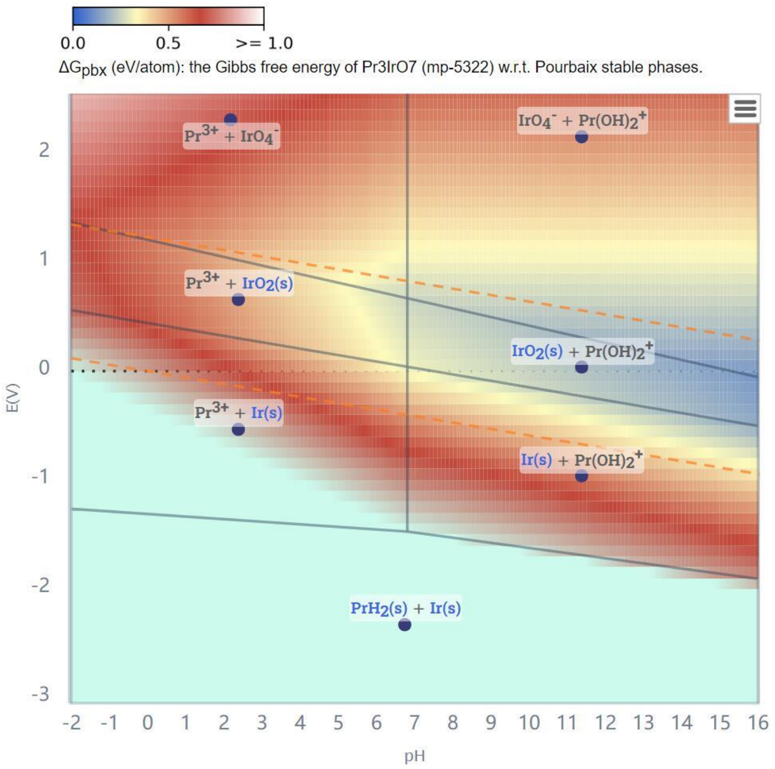

Figure S13. Pourbaix phases diagram of Pr and Ir.

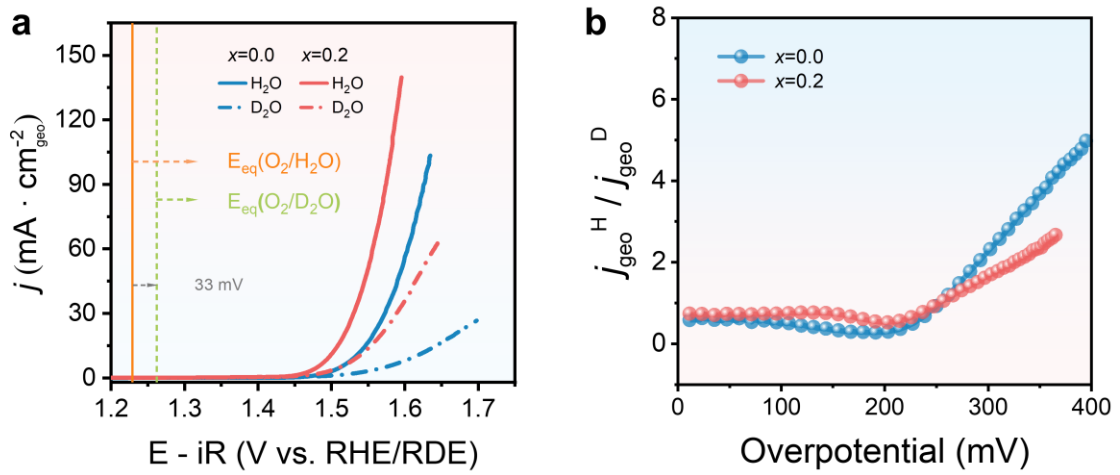

Figure S14. (a) LSV curves for PIO-post and 0.2Mo-PIO-post measured in 0.5 M H_2SO_4 prepared in H_2O and 0.5 M D_2SO_4 prepared in D_2O. (b) KIE of PIO-post and 0.2Mo-PIO-post. j^H and j^D are referred to the current density measured in 0.5 M H_2SO_4 prepared in H_2O and 0.5 M D_2SO_4 prepared in D_2O, respectively.

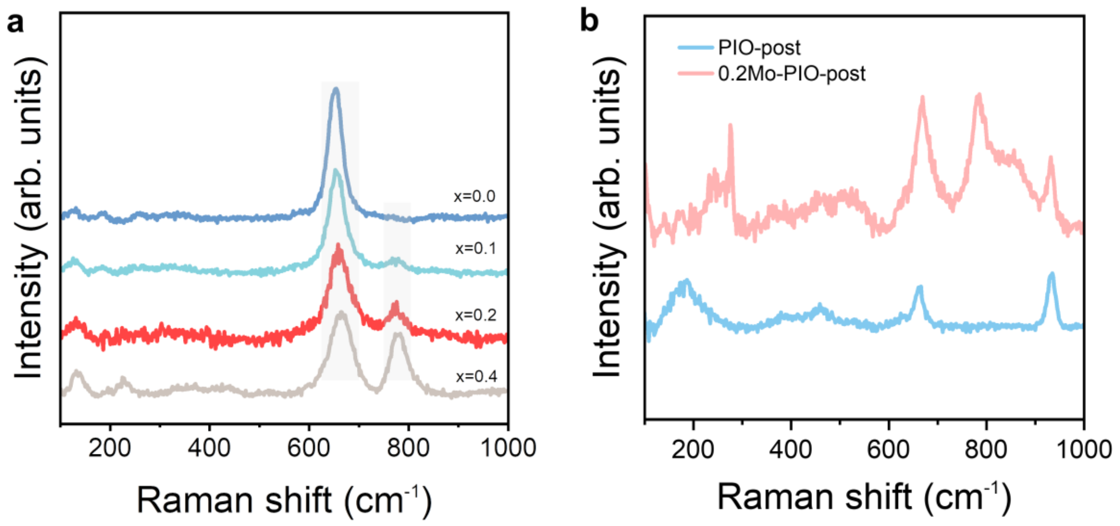

Figure S15. Raman spectra of as-synthesized *x*Mo-PIO (*x* = 0.0, 0.1, 0.2 and 0.4) (a), PIO-post and 0.2Mo-PIO-post (b).

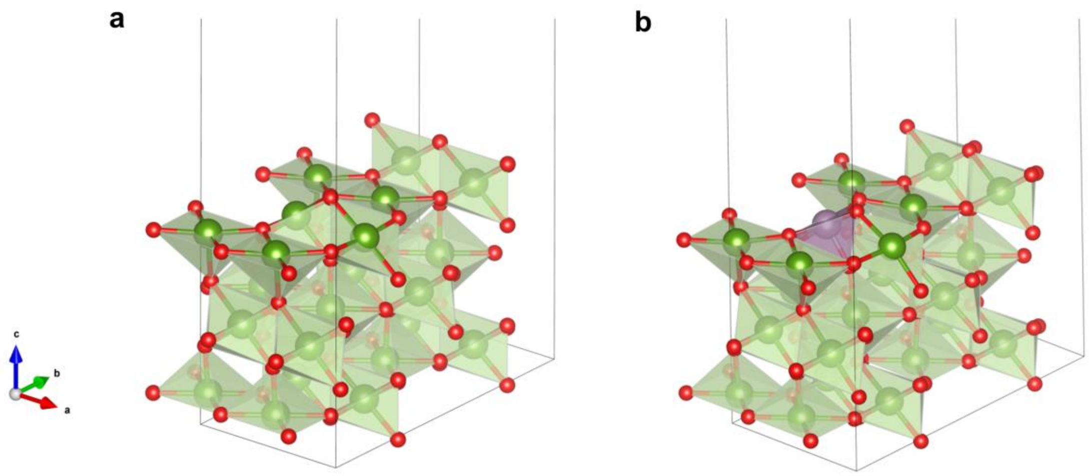

Figure S16. Crystal structure of IrO_2-O_v (a) and Mo doped IrO_2-O_v (b).

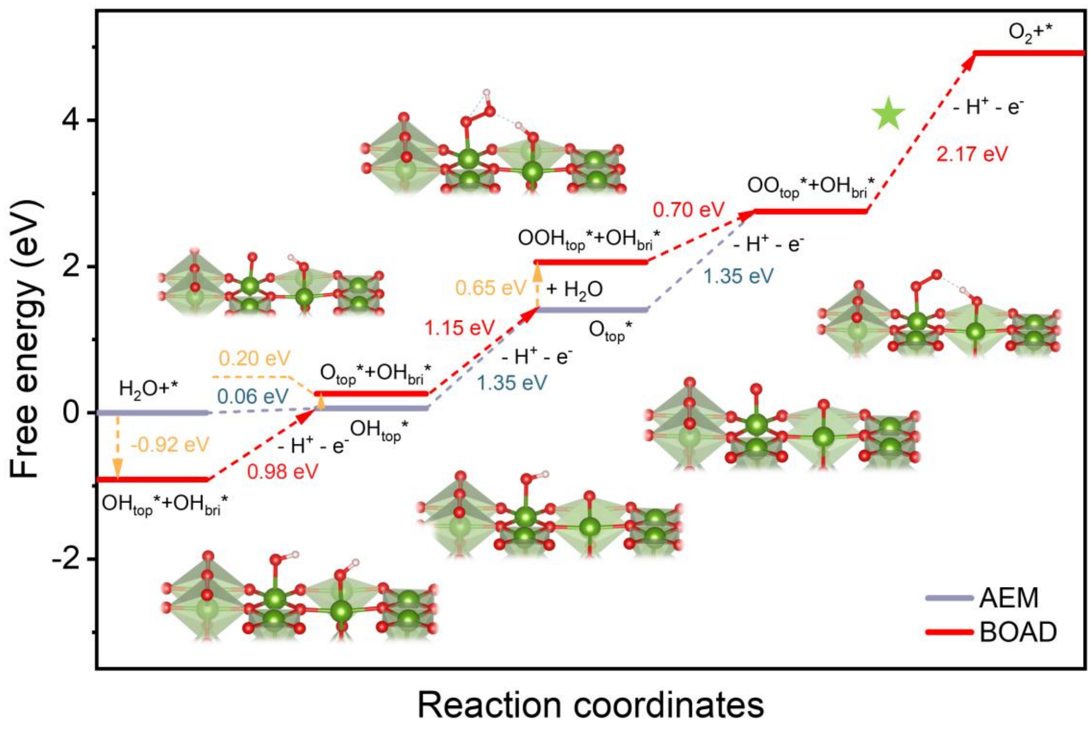

Figure S17. The free energy diagram of IrO_2-O_v with different OER pathways. Color code: Ir (green), O (red). The green octahedra represent IrO_6 octahedra. PDS is labeled by the green star.

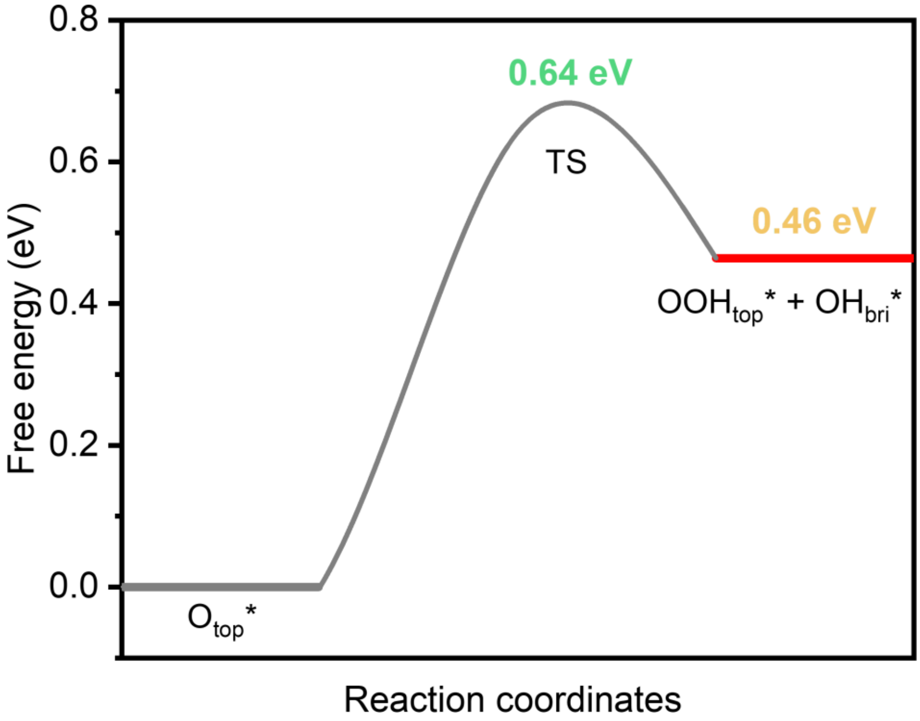

Figure S18. Activation free energy (ΔG_{TS}) for the second H_2O dissociation in BOAD pathway. TS is the transition state, the ΔG_{TS} is 0.64 eV.

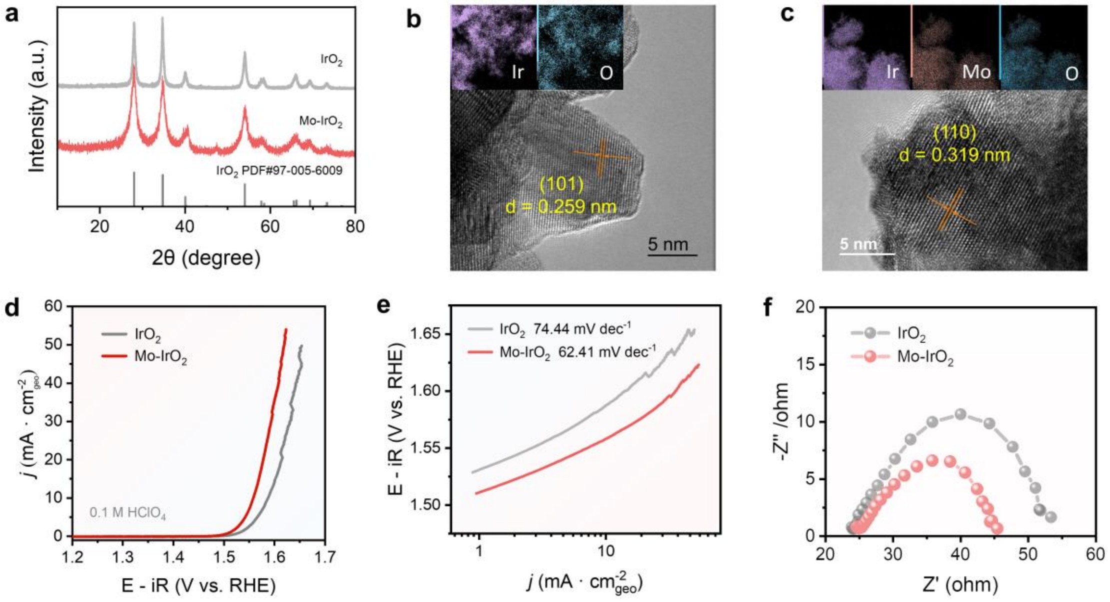

Figure S19. (a) Powder XRD patterns of IrO_2 and Mo-IrO_2. (b), (c) HRTEM images of IrO_2 (b) and Mo-IrO_2 (c). Insets: corresponding EDS elemental mappings of Ir (purple), Mo (orange) and O (blue). (d) Geometric area-normalized LSV curves. (e) Tafel plots based on geometric area-normalized LSV curves. (f) Nyquist plots at 1.55 V_{RHE}.

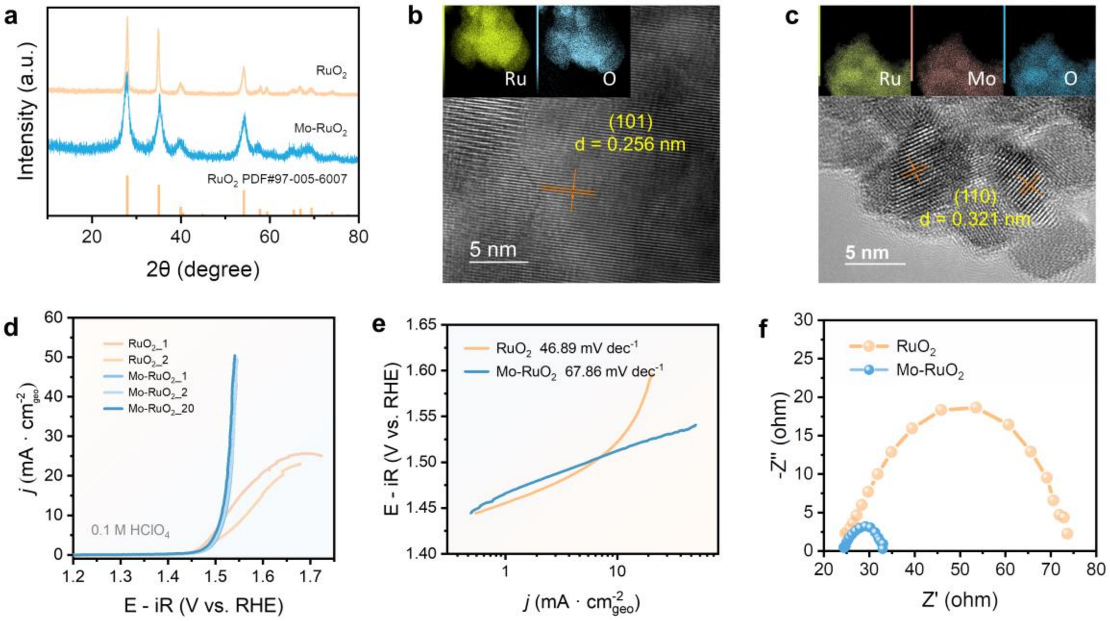

Figure S20. (a) Powder XRD patterns of RuO_2 and Mo-RuO_2. (b), (c) HRTEM images of RuO_2 (b) and Mo-RuO_2 (c). Insets: corresponding EDS elemental mappings of Ru (green), Mo (orange) and O (blue). (d) Geometric area-normalized LSV curves at specific cycles. (e) Tafel plots based on geometric area-normalized LSV curves. (f) Nyquist plots at 1.53 V_{RHE}.

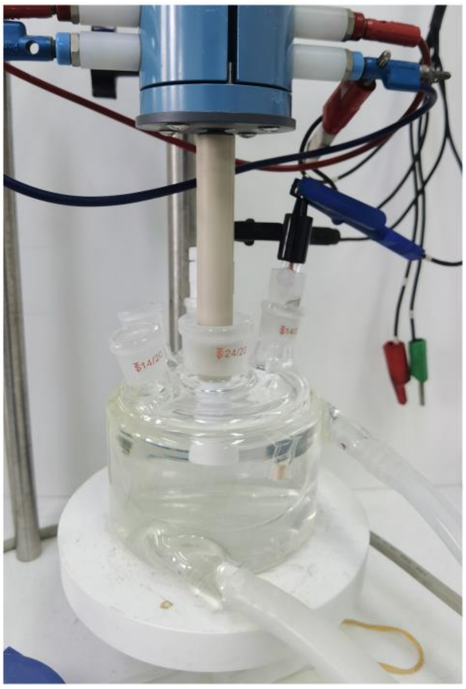

Figure S21. The electrochemical cell set-up.

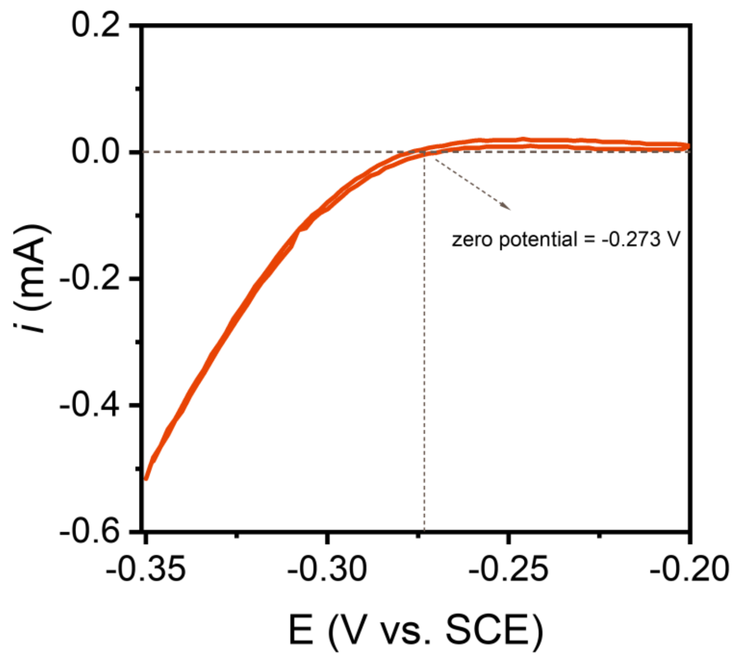

Figure S22. The current as a function of the applied potentials for the calibration of SCE reference electrode.

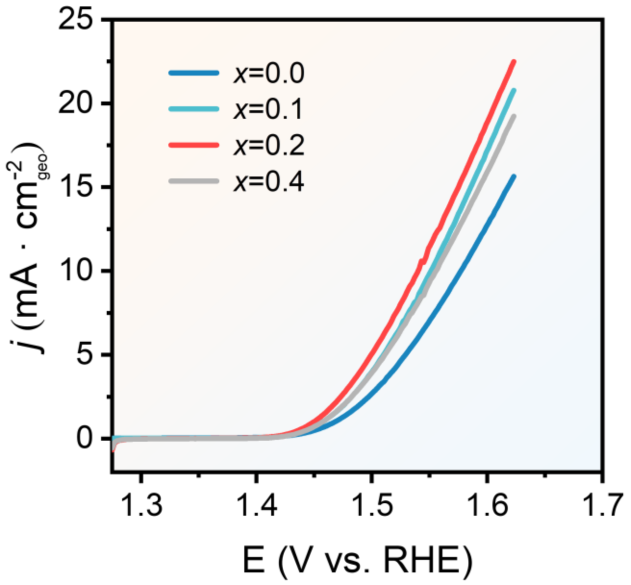

Figure S23. The non-iR corrected LSV curves of OER performance test of *x*Mo-PIO-post (*x* = 0.0, 0.1, 0.2, 0.4).

2. Supplementary Tables

Table S1. Formation energy of different Mo-substitution sites

Lattice site	Formation energy (E_f) / eV
Ir	0.25
$Pr_{(1)}$	7.94
$Pr_{(2)}$	9.57

Table S2. Bader charge analysis

	Pr / |e|	Ir / |e|	Mo / |e|	O / |e|
Pr_3IrO_7	-25.35	-7.02	null	32.37
Mo-Pr_3IrO_7	-25.38	-5.10	-2.38	32.86

Table S3. Band center and charge transfer energy determined by DOS

	$\varepsilon_{O\text{-}p}$ / eV	$\varepsilon_{Ir\text{-}d}$ / eV	$\lvert\varepsilon_{Ir\text{-}d} - \varepsilon_{O\text{-}p}\rvert$ / eV
Pr_3IrO_7	-2.13	-2.45	0.32
Mo-Pr_3IrO_7	-2.21	-2.09	0.12

Table S4. Vacancy formation energy of Pr in Pr_3IrO_7 and Mo-Pr_3IrO_7

	$Pr_{(1)}$ / eV	$Pr_{(2)}$ / eV
Pr_3IrO_7	10.34	10.16
Mo-Pr_3IrO_7	9.92	9.58
Δ	-0.42	-0.58

Table S5. Vacancy formation energy of O in Pr_3IrO_7 and Mo-Pr_3IrO_7

	Ir-$O_{(1)}$ / eV	Mo-$O_{(1)}$ / eV	$O_{(2)}$/ eV	$O_{(3)}$/ eV
Pr_3IrO_7	2.73	null	2.93	2.83
Mo- Pr_3IrO_7	3.02	2.64	3.23	2.66

Ir-$O_{(1)}$ and Mo-$O_{(1)}$ refer to O atoms bonded with Ir and Mo respectively.

Table S6. $Pr_{(2)}$ vacancy formation energy in Pr_3IrO_7 and Mo-Pr_3IrO_7 with/without lattice oxygen vacancies

	$E_{f\text{-}Pr(2)}$ / eV		
	pristine	$O_{(1)\text{-vacancy}}$	$O_{(3)\text{-vacancy}}$
Pr_3IrO_7	10.16	8.79	9.71
Mo- Pr_3IrO_7	9.58	8.50	9.37

Table S7. Ionic radii of Ir and Mo at different oxidation states

Ion	Charge	Coordination	Ionic Radius / Å
Ir	3	VI	0.680
	4		0.625
	5		0.570
Mo	5		0.610
	6		0.590

Table S8. BET surface areas of as-synthesized *x*Mo-PIO

x	Surface area / $m^2\ g^{-1}$
0.0	0.9214
0.1	0.9506
0.2	0.7751
0.4	0.4997

Table S9. Peak area proportion of different Ir oxidation states of as-synthesized *x*Mo-PIO determined by XPS results

x	Ir^{V} (%)	$Ir^{<V}$ (%)	$Ir^{V}/Ir^{<V}$
0.0	68.02	31.98	2.13
0.1	60.41	39.59	1.53
0.2	41.84	58.16	0.72
0.4	25.94	74.06	0.35

Table S10. Peak area proportion of O 1*s* components for as-synthesized *x*Mo-PIO determined by XPS results

x	O_{L1} (%)	O_{L2} (%)	O_{L3} (%)	HO/O_{ads} (%)	H_2O/CO_3^{2-} (%)
0.0	24.50	12.07	22.75	35.17	5.51
0.1	25.07	15.61	28.3	24.85	6.17
0.2	28.58	17.53	33.02	14.96	5.91
0.4	30.27	21.87	25.04	17.95	4.88

Table S11. The comparison of OER performance with some representative Ir-based electrocatalysts in acidic media.

Catalysts	Electrolytes	Mass loading	Mass activity	Ref.
0.2Mo-PIO-post	0.1 M $HClO_4$	0.25 mg·cm^{-2}	415 A·g_{Ir}^{-1}@1.52 V	This work
PIO-post	0.1 M $HClO_4$	0.25 mg·cm^{-2}	97 A·g_{Ir}^{-1}@1.52 V	This work
IrO_2	0.1 M $HClO_4$	0.25 mg·cm^{-2}	4.7 A·g_{Ir}^{-1}@1.52 V	This work
$Pt_{39}Ir_{10}Pd_{11}$	0.1 M $HClO_4$	16.8 μg $_{Pt+Ir+Pd}$·cm^{-2}	200 A·$g_{Pt+Ir+Pd}^{-1}$@1.53V	*Adv. Energy Mater.* **10**, 1904114 (2020).
9R-$BaIrO_3$	0.5 M H_2SO_4	0.28 mg·cm^{-2}	168 A·g_{Ir}^{-1}@1.5 V	*J. Am. Chem. Soc.* **143**, 43, 18001 (2021).
P-$IrCu_{1.4}$ NCs	0.05 M H_2SO_4	60 μg $_{Ir}$·cm^{-2}	213 A·g_{Ir}^{-1}@1.55 V	*Chem. Mater.* **30**, 8571 (2018).
6H-$SrIrO_3$	0.5 M H_2SO_4	0.9 mg·cm^{-2}	75 A·g_{Ir}^{-1}@1.525 V	*Nat. Commun.* **9**, 5236 (2018).
IrNiCu DNF/C	0.1 M $HClO_4$	20 μg·cm^{-2}	460 ± 70 A·g_{Ir}^{-1} @1.53 V	*ACS Nano* **11**, 5500 (2017).
cobalt-doped 6H-$SrIrO_3$	0.1 M $HClO_4$	0.45 mg·cm^{-2}	286.7 A·g_{Ir}^{-1}@1.55 V	*ACS Appl. Mater. Interfaces* **11**, 42006 (2019).
$Y_2[R_{u1.6}Y_{0.4}]O_{7-\delta}$	0.1 M $HClO_4$		150 A·g^{-1}@1.45 V	*Angew. Chem. Int. Ed.* **130**, 14073 (2018).
$Pr_2Ir_2O_7$	0.1 M $HClO_4$	0.057 mg·cm^{-2}	424.5 A·g_{Ir}^{-1}@1.53 V	*Adv. Mater.* **31**, 1805104 (2019).
Amorphous Ir nanosheets	0.1 M $HClO_4$	0.2 mg·cm^{-2}	221.8 A·g^{-1}@1.53 V	*Nat. Commun.* **10**, 4855 (2019).
Co-doped IrCu	0.1 M $HClO_4$	25.5 μg·cm^{-2}	640 A·g_{Ir}^{-1}@1.53 V	*Adv. Funct. Mater.* **27**, 1604688 (2017).
Pd@Ir_{3L}	0.1 M $HClO_4$	10.2 μg $_{Ir}$·cm^{-2}	333 A g $_{Ir}^{-1}$@1.53 V	*Chem. Mater.* **31**, 5867 (2019).
Ba_2PrIrO_6	0.1 M $HClO_4$	0.95 mg·cm^{-2}	10 A g^{-1}@1.5 V	*Nat. Commun.* **7**, 12363 (2016).
IrO_2 Nanoneedles	1 M H_2SO_4	0.25 mg·cm^{-2}	50 A·g_{Ir}^{-1}@1.55 V	*Adv. Funct. Mater.* **28**, 1704796 (2018).

Table S12. The comparison of OER performance with some representative acidic OER electrocatalysts in 0.5 M H_2SO_4.

Catalysts	η_{10} (mV)	Mass loading	Mass activity	Tafel slope (mV·dec^{-1})	Ref.
0.2Mo-PIO-post	265	0.25 mg·cm^{-2}	1216 A·g_{Ir}^{-1}@1.55V	62.15	This work
PIO-post	303	0.25 mg·cm^{-2}	308A·g_{Ir}^{-1}@1.55V	76.68	This work
IrO_2	337	0.25 mg·cm^{-2}	33 A·g_{Ir}^{-1}@1.55V	70.21	This work
W-Ir-B alloy	300	78.9 μg·cm^{-2}	518 A·g_{Ir}^{-1}@1.53V	78	*Nat. Commun.* **12**, 3540 (2021).
AlNiCoIrMo np-HEA (20 wt%)	275		115 A g^{-1}@1.5 V	55.2	*Small* **15**, 1904180 (2019).
Li-IrO_x	290	0.125 mg·cm^{-2}	100 A·g_{Ir}^{-1}@1.52V	39	*J. Am. Chem. Soc.* **141**, 3014 (2019).
Ru-N-C	267	0.28 mg·cm^{-2}	3571 A·g_{Ru}^{-1}@1.497 V	52.6	*Nat. Commun.***10**, 4849 (2019).
Co_3O_4/CeO_2	423			88.1	*Nat Commun* **12**, 3036 (2021).
Ir/Fe_4N	316 ± 5	76.5 μg·cm^{-2}	116.4 mA·μg_{Ir}^{-1} @1.54 V	61.5	*ACS Catal.* **8**, 2615 (2018).
$Rh_{22}Ir_{78}$ NPs	292 ± 1	0.28 mg·cm^{-2}	1174 ±20 A·g_{Ir}^{-1}@1.53 V	101	*ACS Nano* **13**, 13225 (2019).
$IrO_x/SrIrO_3$	270-290				*Science* **353**, 1011 (2016).
Ir@N-G-750	303	23 μg·cm^{-2}	2420 A·g^{-1}@1.6 V	50	*Nano Energy* **62**, 117 (2019).
IrO_2@Ir/TiN	265	0.379 mg·cm^{-2}	480.4 A·g_{Ir}^{-1}@1.6 V	52.3	*ACS Appl. Mater. Interfaces* **10**, 38117 (2018).
6H-$SrIrO_3$	248	0.9 mg·cm^{-2}	75 A·g_{Ir}^{-1}@1.525 V		*Nat. Commun.* **9**, 5236 (2018).
TiN/IrO_2	313		874 A $g_{IrO_2}^{-1}$@1.6 V	65.5	*J. Mater. Sci.* **55**, 3507 (2020).

Table S13. Rough factors of *x*Mo-PIO-post

x	Rough factor	ECSA / cm^2
0.0	22.16	4.343
0.1	37.66	7.381
0.2	38.83	7.611
0.4	52.66	10.321

Table S14. Tafel slopes of *x*Mo-PIO-post

	Tafel slope / mV dec^{-1}	
x	ECSA-normalized	Geometric area-normalized
0.0	66.93	70.22
0.1	56.28	55.37
0.2	50.42	50.52
0.4	60.37	59.19

Table S15. Fitting results of Nyquist plots for *x*Mo-PIO-post

x	0.0	0.1	0.2	0.4
R_s (Ω)	24.71	24.26	25.15	25.01
R_{ct} (Ω)	22.67	15.95	6.709	10.23
CPE-T	0.0010591	0.0024859	0.0045267	0.0042261
CPE-P	0.72358	0.70772	0.84393	0.6627

R_s is the solution resistance, R_{ct} is the charge transfer resistance, and CPE is the constant phase element.

Table S16. The comparison of stability number with some representative Ir-based electrocatalysts in acidic media.

Catalysts	Electrochemical testing conditions	Electrolyte	S-number	Ref.
0.2Mo-PIO-post	20 mA cm^{-2} , 140 h	0.1 M $HClO_4$	2.1×10^8	This work
commercial IrO_2	20 mA cm^{-2} , 20 h	0.1 M $HClO_4$	6.5×10^7	This work
IrO_x	5 mV s^{-1} sweep to 1.65 V_{RHE}	0.1 M $HClO_4$	5.7×10^4	*Nat. Catal.* **1**, 508 (2018).
$SrIrO_3$	5 mV s^{-1} sweep to 1.65 V_{RHE}	0.1 M $HClO_4$	1.8×10^4	*Nat. Catal.* **1**, 508 (2018).
$SrIr_{0.8}Zn_{0.2}O_3$	1.7 V_{RHE}, 160 min	0.1 M $HClO_4$	3.2×10^5	*ACS Appl. Energy Mater* **5**, 12206 (2022).
IrO_x	100 A/g_{Ir}, 2 h	0.1 M H_2SO_4	6.0×10^4	*Nat. Commun.* **12**, 1 (2021).
porous IrO_x-500 °C	10 mA cm^{-2} , V_{cutoff} = 2 V_{RHE}	0.05 M H_2SO_4	3.5×10^4	*ACS Catal.* **11**, 4107 (2021).
$SrCo_{0.9}Ir_{0.1}O_{3-\delta}$	10 mA cm^{-2} , 3 h	0.1 M $HClO_4$	8.6×10^4	*Nat. Commun.* **10**, 1 (2019).
$SrIrO_3$|STO	1.7 V_{RHE} average, 6 h	0.5 M H_2SO_4	9.8×10^3	*Adv. Funct. Mater.* **31,** 2101542 (2021).
BCC-Cr-$SrIrO_3$	1.53 V_{RHE}, 40 h	0.1 M $HClO_4$	6.5×10^5	*Nano Energy* **102**, 107680 (2022).
3C-$SrIrO_3$	1.53 V_{RHE}, 40 h	0.1 M $HClO_4$	2.5×10^4	*Nano Energy* **102**, 107680 (2022).
6H-$SrIrO_3$	1 mA cm^{-2} , 1 h	0.1 M $HClO_4$	2.2×10^4	*Chem. Mater.* **32**, 3499 (2020).
$Y_2Ir_2O_7$	1.6 V_{RHE}, ~2.5 h	0.5 M H_2SO_4	5.5×10^3	*J. Phys. Chem. C* **126**, 1751 (2022).
Ir/$CuTiON_x$/C	5 mA cm^{-2} , 5 min	0.1 M $HClO_4$	7.8×10^4	*ACS Catal.* **11**, 12510 (2021).
$Re_{0.1}$-IrO2	10 mA cm^{-2} , 30 h	0.5 M H_2SO_4	3.7×10^{10}	*Small* 2207847 (2023).

Table S17. Element compositions determined by EDS results of 0.0Mo-PIO at varied electrochemical cycles

Number of cycles	Element	Mass %	Atom %	Pr / Ir
2	O K	17.38	67.56	1.94
	Pr L	48.60	21.44	
	Ir M	34.02	11.00	
10	O K	14.68	63.67	1.63
	Pr L	45.69	22.51	
	Ir M	38.26	13.82	
20	O K	15.84	65.86	1.22
	Pr L	39.76	18.77	
	Ir M	44.41	15.37	

Table S18. Element compositions determined by EDS results of 0.2Mo-PIO at varied electrochemical cycles

Number of cycles	Element	Mass %	Atom %	Pr / (Ir + Mo)
2	O K	22.15	72.88	1.86
	Mo L	3.93	2.16	
	Pr L	47.24	17.65	
	Ir M	26.68	7.31	
10	O K	15.37	63.86	1.43
	Mo L	3.45	2.39	
	Pr L	45.10	21.27	
	Ir M	36.07	12.47	
20	O K	16.48	65.94	0.944
	Mo L	5.47	3.65	
	Pr L	36.40	16.54	
	Ir M	41.65	13.87	

3. Supplementary Notes

Supplementary Note 1

Ln_3IrO_7 (Ln = Pr, Nd, Sm, Eu) is a superstructure of the cubic fluorite structure with space group *Cmcm*.[1, 2, 3] The structure features three different O sites and two distinctive Ln sites. Corner-linked IrO_6 octahedra lie along *c*-axis with shared apical $O_{(3)}$. One third of Ln ($Ln_{(1)}$) are in eight-fold oxygen coordination (four $O_{(1)}$ and four $O_{(2)}$), forming edge-shared $Ln_{(1)}O_8$ cubes alternatively parallel to IrO_6 chains. $Ln_{(1)}O_8$ cubes chains and IrO_6 octahedra chains share two equatorial $O_{(1)}$, forming slabs aligned with *bc*-plane. The remaining $Ln_{(2)}$ are in seven-fold oxygen coordination (four $O_{(1)}$, two $O_{(2)}$ and one $O_{(3)}$), lie between slabs forming by $Ln_{(1)}O_8$ cubes chains and IrO_6 octahedra chains.

Supplementary Note 2

The peaks located at about 528.7 eV and 529.8 eV can be assigned as two kinds of lattice oxygen (O_{L1} and O_{L2}) with the former being characteristic of Pr–O bond and the latter corresponding to highly oxidized surface lattice oxygen species (O_2^{2-}/O^-). The ones at around 531.3 eV and 532.9 eV correspond to surface hydroxyl or adsorbed oxygen species ($-OH/O_{ads}$) and adsorbed water molecules or carbonates (H_2O/CO_3^{2-}) respectively.[4, 5]

Supplementary Note 3

As supported by reported theory and experimental evidence, the main line peaks of Ir^{III} species appear at around 62.3 eV and 65.3 eV for Ir $4f_{7/2}$ and Ir $4f_{5/2}$, respectively, which show positive shift of binding energies compared with those for Ir^{IV} species (around 61.7 eV and 64.7 eV for Ir $4f_{7/2}$ and Ir $4f_{5/2}$, respectively). As a result, although the peak of $Ir^{<V}$ species is located at the lower energy compared to that of Ir^{III} species, the average oxygen state is above +3. After 2 electrochemical cycles, the surface lattice oxygen oxidation (O_2 release) and subsequent occupation of oxygen vacancies with water molecules or hydroxyl contribute to the reduction of metal oxidation state. As shown in HRTEM and corresponding SAED images (Figure 4a, b right panel), sparse particles appear on the surface but with well-ordered bulk crystalline, indicating an ongoing surface reconstruction. So, the surface is not fully covered with hydroxide species IrO_x.

Supplementary Note 4: Calculation of the mole fraction of ^1H in all hydrogen

We calculated the mole fraction of ^1H in all hydrogen to elucidate that ^1H in

nondeuterated $HClO_4$ will not affect the results of the experiment significantly.

Take 1 L 0.1 M $HClO_4$ (in D_2O) as an example.

The total amount of ^1H is 0.1 mole. The volume of D_2O used is

$$1000\ \text{mL} - 8.625\ \text{mL} = 991.375\ \text{mL} \qquad (1)$$

where 8.625 mL is the volume of $HClO_4$ added.

The total amount of D is

$$991.375\ \text{mL} \times 1.1056\ \text{g mL}^{-1} \div 20.0276\ \text{g mol}^{-1} \times 2 = 109.4554\ \text{mol} \qquad (2)$$

where 1.1056 g mL^{-1} is the density of D_2O, 20.0276 g mol^{-1} is the molecular weight of D_2O.

So the mole fraction of ^1H is

$$\frac{0.1\ \text{mol}}{0.1\ \text{mol} + 109.4554\ \text{mol}} \times 100\% = 0.0913\% \qquad (3)$$

The mole fraction of ^1H is so small that it has negligible effect on the experiment. So $HClO_4$ was used for KIE analysis.

Supplementary Note 5

Although group-theoretical analysis for the Γ-point phonon modes in Ln_3MO_7 (*Cmcm*) expects 27 ($8A_g + 8B_g + 5B_{2g} + 6B_{3g}$) Raman active modes, only 7 A_g bands (~134 cm^{-1}, ~185cm^{-1}, ~259 cm^{-1}, ~313 cm^{-1}, ~342 cm^{-1}, ~586 $cm^{-1}$and ~653 cm^{-1}) are clearly identified here because some occur beyond detecting limit (below 100 cm^{-1}) or too much lower to be observed.[6, 7, 8] Raman bands for Mo-doped samples resemble those observed in 0.0Mo-PIO except the obvious presence of a new band at around 770 cm^{-1} which is more intense with the increment of Mo-doping concentration. Frequency of this new Raman signal is in accordance with that of the high-frequency phonon band of MoO_6 octahedra with A_g symmetry. Therefore, it is reasonable to attribute the new band to Mo–O vibration mode with the substitution of Mo for Ir in [IrO_6] octahedra (Figure S15a).

Prior to in situ Raman measurements, LSV was conducted for both 0.0Mo-PIO and 0.2Mo-PIO to reach steady-state polarization curves. Bands at ~134 cm^{-1}, ~259 cm^{-1}, ~313 cm^{-1}, ~342 cm^{-1}, ~586 cm^{-1} for pristine samples have vanished after reconstruction, indicating the significantly destroyed chemical structures when suffering oxidizing potentials, which is consistent with the results from TEM and XPS analysis. Meanwhile, peaks are broader and weaker with obvious frequency shift, indicating relatively poor crystallinity and changed local coordination structures of newly formed surface layers. (Figure S15b)

Supplementary Note 6

In the transition state theory (TST), it is generally acknowledged that the TS locates at a point of no return between reactant state and product. And the transition of reactant to TS is the rate determining step (RDS).[9] Based on this, the rate constant can be deduced as:

$$k_{TST} = \frac{k_B T}{h} e^{-\Delta G_{TS}/k_B T} \qquad (4)$$

in which ΔG_{TS} is the difference of Gibbs energy between TS and the reactant state. This equation is the formula used in TST to calculate the rate constant of a reaction using thermodynamic methods. It can be applied to elementary reaction to calculate the reaction's rate constant so long as the activation free energy (ΔG_{TS}) can be obtained. And the rate constant is equal to the reaction rate. As a rule of thumb, a reaction rate on the order of 1 site^{-1} s^{-1} should be attained for a reasonable catalyst. On the base of 1 site^{-1} s^{-1}, Nørskov et al. depicted the relation of the ΔG_{TS} and the temperature (T) as shown in Figure S24.[9] It reveals that the reaction rate of 1 site^{-1} s^{-1} corresponds to an ΔG_{TS} of 0.75 eV at room temperature (300 K). As a consequence, the activation free energy of 0.75 eV can be considered as a limit barrier for the chemical reaction steps at room temperature. Then we calculated the free energy barrier (ΔG) of the chemical step: O_{top}* +H_2O → OOH_{top}* + OH_{bri}*. The corresponding ΔG_{TS} is 0.64 eV, which below 0.75 eV, indicating the kinetic barrier of chemical steps associated with the BOAD pathway can be readily overcome at room temperature.

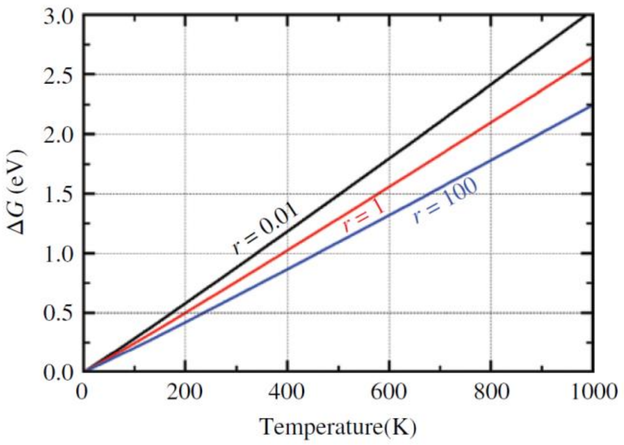

Figure S24. The Gibbs free energy of activation, ΔG, plotted as a function of temperature for r = 0.01 s^{-1} (black curve), r = 1 s^{-1} (light gray curve), and r = 100 s^{-1} (dark gray curve) as calculated using equation (4).[9] Copyright 2014, John Wiley & Sons.

Supplementary Note 7

As shown in Figure S19a, powder XRD patterns of as synthesized IrO_2 and Mo-IrO_2 can be well indexed to rutile IrO_2 (PDF # 97-005-6009). HRTEM of IrO_2 (Figure S19b) and Mo-IrO_2 (Figure S19c) confirm the good crystallization. The interplanar spacing of about 0.259nm in Figure S18b and 0.319 nm in Figure S19c coincide well with (101) and (110) facets of rutile IrO_2 respectively. Besides, no impurity particles are observed in Mo-IrO_2 and EDS elemental mapping results (insets) clearly show the homogeneous dispersion of Mo in the lattice. These results demonstrate that Mo atoms are successfully introduced into IrO_2 lattice with well-maintained crystallization. OER performance was assessed in 0.1 M $HClO_4$ electrolyte under ambient conditions. Mo-IrO_2 exhibits an overpotential of 320 mV to reach 10 mA cm_{geo}^{-2}, lower than that of IrO_2 (350 mV) (Figure S19d). Tafel slope of Mo-IrO_2 (62.41 mV dec^{-1}) is smaller than undoped IrO_2 (74.44 mV dec^{-1}), suggesting the optimized kinetics of IrO_2 after Mo substitution (Figure S19e). The decreased semicircles in Nyquist plots (Figure S19f) clearly demonstrate a facilitated charge transfer process of Mo-IrO_2. These results manifest that Mo can be successfully introduced into IrO_2 system and indeed has a positive effect during acidic OER.

Supplementary Note 8

As shown in Figure S20a, powder XRD patterns of as synthesized RuO_2 and Mo-RuO_2 can be well indexed to rutile RuO_2 (PDF # 97-005-6007). HRTEM of RuO_2 (Figure S20b) and Mo-RuO_2 (Figure S20c) confirm the good crystallization. The interplanar spacing of about 0.256 nm in Figure S19b and 0.321 nm in Figure S20c coincide well with (101) and (110) facets of rutile RuO_2 respectively. Besides, no impurity particles are observed in Mo-RuO_2 and EDS elemental mapping results (insets) clearly show the homogeneous dispersion of Mo in the lattice. These results demonstrate that Mo atoms are successfully introduced into RuO_2 lattice with well-maintained crystallization. OER performance assessment in 0.1 M $HClO_4$ demonstrates that Mo has significant influence on the behavior of RuO_2. The activity of RuO_2 deteriorates quickly after one LSV because of the excessive oxidation of Ru species at high potentials. While the polarization curves of Mo-RuO_2 exhibit no decay after 20 cycles (Figure S20d), suggesting the improved stability upon Mo substitution. Mo-RuO_2 requires an overpotential of 290 mV to reach 20 mA cm_{geo}^{-2}, lower than that of RuO_2 (360 mV). Tafel slope of Mo-RuO_2 (46.89 mV dec^{-1}) is smaller than undoped RuO_2 (67.86 mV dec^{-1}), suggesting the optimized kinetics (Figure S20e). The decreased semicircles in Nyquist plots (Figure S20f) clearly demonstrate a

facilitated charge transfer process of Mo-RuO_2. Consequently, Mo doping not only contributes to higher OER activity but importantly, the excessive oxidation at higher potentials of Mo-RuO_2 has been obviously impeded, which is more important for practical applications.

References

1. Vente J, IJdo D. The orthorhombic fluorite related compounds Ln_3IrO_7. *Mater. Res. Bull.* **26**, 1255-1262 (1991).
2. Nishimine H, Doi Y, Hinatsu Y, Sato M. Phase transition of Ln_3IrO_7 (Ln = Pr, Nd, Sm, Eu) and its low-temperature structure. *J. Ceram. Soc. Jpn.* **115**, 577-581 (2007).
3. Qin Q, *et al.* Gettering La effect from La_3IrO_7 as a highly efficient electrocatalyst for oxygen evolution reaction in acid media. *Adv. Energy Mater.* **11**, 2003561-2003568 (2020).
4. Xu X, *et al.* A perovskite electrocatalyst for efficient hydrogen evolution reaction. *Adv. Mater.* **28**, 6442-6448 (2016).
5. Diaz-Morales O, *et al.* Iridium-based double perovskites for efficient water oxidation in acid media. *Nat. Commun.* **7**, 12363-12368 (2016).
6. Bontchev R, *et al.* Crystal structure, electric and magnetic properties, and Raman spectroscopy of Gd_3RuO_7. *Phys. Rev. B* **62**, 12235-12240 (2000).
7. Siqueira KPF, *et al.* Crystal structure of fluorite-related Ln_3SbO_7 (Ln = La–Dy) ceramics studied by synchrotron X-ray diffraction and Raman scattering. *J. Solid State Chem.* **203**, 326-332 (2013).
8. Siqueira KPF, *et al.* Synchrotron X-ray diffraction and Raman spectroscopy of Ln_3NbO_7 (Ln = La, Pr, Nd, Sm-Lu) ceramics obtained by molten-salt synthesis. *J. Solid State Chem.* **209**, 63-68 (2014).
9. Nørskov JK, Studt F, Abild-Pedersen F, Bligaard T. *Fundamental concepts in heterogeneous catalysis*. John Wiley & Sons (2014).
