# Supplementary material for: Reconstructed Ir‒O‒Mo species with strong Brønsted acidity for acidic water oxidation
Source: Nat Commun. 2023 Jul 12;14:4127. doi: 10.1038/s41467-023-39822-6 (PMC10338439; doi:10.1038/s41467-023-39822-6)
Supplement: Supplementary file 3 — Description of Additional Supplementary Files [file 41467_2023_39822_MOESM3_ESM.pdf]

Description of Additional Supplementary Files

Supplementary Data: POSCAR files of Pr_3IrO_7, Mo-Pr_3IrO_7, IrO_2-O_v and Mo-IrO_2- O_v
